# Intestinal Morphometry, Enzymatic and Microbial Activity in Laying Hens Fed Different Levels of a *Hermetia illucens* Larvae Meal and Toxic Elements Content of the Insect Meal and Diets

**DOI:** 10.3390/ani9030086

**Published:** 2019-03-10

**Authors:** Giuseppe Moniello, Andrea Ariano, Valentina Panettieri, Francesca Tulli, Ike Olivotto, Maria Messina, Basilio Randazzo, Lorella Severino, Giovanni Piccolo, Nadia Musco, Nicola Francesco Addeo, Georges Hassoun, Fulvia Bovera

**Affiliations:** 1 Department of Veterinary Medicine, University of Sassari, Via Vienna 2, 07100 Sassari, Italy; moniello@uniss.it; 2 Department of Veterinary Medicine and Animal Production, University of Napoli Federico II, via F. Delpino 1, 80137 Naples, Italy; andrea.ariano@unina.it (A.A.); valentina.panettieri@unina.it (V.P.); lorella.severino@unina.it (L.S.); nadia.musco@unina.it (N.M.); nicolafrancesco.addeo@unina.it (N.F.A.); bovera@unina.it (F.B.); 3 Department of AgriFood, Environment and Animal Science, University of Udine, via Sondrio, 2, 33100 Udine, Italy; francesca.tulli@uniud.it (F.T.); maria.messina@uniud.it (M.M.); 4 Department of Life and Environmental Sciences, University Politecnica delle Marche, via Brecce Bianche, 60131 Ancona, Italy; i.olivotto@staff.univpm.it (I.O.); b.randazzo@staff.univpm.it (B.R.); 5 Faculty of Agricultural Engineering and Veterinary Medicine, Lebanese University, Beirut, Lebanon; ghassoun@ul.edu.lb

**Keywords:** *Hermetia illucens* larvae meal, volatile fatty acids, intestinal villi height and villi/crypt ratio, brush border enzymes, intestinal alkaline phosphatase, trace elements, laying hens

## Abstract

**Simple Summary:**

Recently, several studies have focused on the use of insect larvae meal as an alternative to soybean meal in poultry diets. In this regard, it is crucial to understand all the possible aspects related to the chemical-nutritional characteristics, the effects on the animals’ health and welfare, and the safety of different insect meals. This study aimed to evaluate volatile fatty acids production in the caeca, the intestinal morphometry, and the brush border enzymatic activity of hens fed different levels of *Hermetia illucens* (Linnaeus) (Diptera: Stratiomyidae) larvae meal for 24 weeks. The research also aimed to contribute to the knowledge of the concentration of toxic elements in insect meals. Overall, the insect meal inclusion affected the small intestine morphometry, the enzymatic activity, and the caecal microbial activity. The soybean meal group showed the highest intestinal functionality, while a compensatory mechanism, probably mediated by the chitin, led to a positive increase of volatile fatty acids and butyrate in the 50% protein replacement diet with potential positive effects on gut healthiness. The levels of toxic elements in the diets and insect meal were lower than the maximum levels of heavy metals set by the EU Commission for the feed.

**Abstract:**

To evaluate the effects of feeding a *Hermetia illucens* (HI) larvae meal on the different intestinal traits of hens, and to determine the toxic elements’ concentration in the insect meal and diets, 162 hens were randomly allotted to three groups. The control received a corn-soybean meal-based diet (SBM); the HI25 and HI50 groups received two diets in which the 25% and 50% of the dietary protein were replaced by the HI protein, respectively. The duodenal and jejunal villi height and villi/crypt were higher (*p* < 0.01) in the SBM than in the HI groups. The ileal villi height was higher (*p* < 0.05) in the SBM and HI25 groups than the HI50. The HI50 group exhibited a lower duodenal maltase activity. The intestinal alkaline phosphatase (IAP) activity linearly decreased in the duodenum and jejunum as the dietary insect meal inclusion increased. The HI50 group had a higher acetate and butyrate level than the SBM. The levels of cadmium (Cd), lead (Pb), mercury (Hg), and arsenic (As) in the diets and insect meal were lower than the maximum values established by the EU Commission. The 25% soybean protein replacement with *Hermetia illucens* larvae meal in the diet of laying hens was more suitable and closer to the optimal level than 50%.

## 1. Introduction

The EU approval for the use of the insect meals in poultry nutrition is “not so distant in the future”, and the approval by the EU Member States could be possible during the first quarter of 2019 [1,2]. Thus, it is mandatory to understand all the possible aspects related to the chemical-nutritional characteristics, the effects on the animal health and welfare, the impact of feed and food safety of the different meals deriving from insects. This goal is not easy to reach as some characteristics can be modified according to the species and, within the species, in relation to the harvesting stage, the growth substrate, etc. In recent years, several studies pointed out the attention on the use of insect meals in growing broiler [3,4,5,6], quail [7], and barbary partridge [8,9]. In laying hens, the available literature on the effects of insect meals on laying performance, egg quality, metabolic and nutritional effects is limited and very often the results are conflicting due to the different species of insects used, the different strains and age of lay of the hens utilized in the trials [10,11,12,13].

An important aspect concerning the use of insect meal as a feed ingredient, related to human and animal health, is the possible accumulation of mineral elements in the insect body during the growing cycle. Some elements (Cu, Se, Cr, Fe, Mn, Ni) are essential for biological functions, but the heavy metals like Cd, Pb, Hg, and As can induce adverse effects due to their potential toxicity and bioaccumulation in the food chain [14]. To regulate animal dietary exposure to heavy metals, the EU Commission established maximum levels (MLs) for different undesirable substances in animal feeds [15]. Data on the transfer of chemical contaminants from different substrates to the insects are very limited [16]. Therefore, monitoring of toxic elements concentrations, in the insect meal and diets, is necessary from the point of view of both nutrition and contamination. The few studies on the mineral profile of insect meals [7,17,18] often showed different minerals with a very wide range of variation according to the composition of the substrate used for the insects growing. For example, increasing the inclusion level of the brown algae, *Ascophyllum nodosum*, in the growing substrate led to increased calcium, sodium, and magnesium and decreased phosphorus, manganese, and copper concentrations in *Hermetia illucens* larvae [17].

This study represents the continuation and completion of a previous trial [12] in which the laying performance, blood profiles, and nutrient digestibility of hens fed *Hermetia illucens* (HI) larvae meal from 16 to 40 weeks of age were investigated. Thus, the aim of the present research was to evaluate the effect of the inclusion of a partially defatted meal from HI larvae in the diet on the volatile fatty acids (VFAs) production in the caeca, intestinal morphometry, and brush border enzymatic activity of 40 weeks old layers. In addition, the research aimed to contribute to the knowledge of the trace and toxic elements’ concentration in insect meals.

## 2. Materials and Methods 

### 2.1. Animals and Experimental Treatments

All the animals were treated according to the principles of the animal welfare stated by the EC Directive 63/2010/EEC regarding the protection of the animals used for experimental and other scientific purposes. The experimental procedures were approved by the Ethical Animal Care and Use Committee of the Department of Veterinary Medicine and Animal Production of the University of Napoli Federico II, Italy (prot. N. 2017/0017676).

The trial was carried out in a private laying hens farm located in Sardinia (Italy) for 24 weeks, from February to July 2017.

A total of 162, sixteen weeks old Hy-line Brown hens (average live weight 1.41 kg ± 0.13 standard deviation) were equally divided into three experimental groups, differing for the dietary treatment. The control group was fed a corn-soybean meal-based diet (SBM group) formulated to meet the hens’ nutrients requirements according to the Hy-line Brown commercial line management guide 2016 [19]. In the other groups, the soybean meal was partially replaced by a partially defatted insect meal obtained from *Hermetia illucens* larvae (HI, Hermetia Deutschland GmbH & Co KG, Amtsgericht Potsdam, Germany) in order to formulate two isoproteic and isoenergetic diets. In the HI25 group, the 25% of the dietary protein was replaced by the HI protein (inclusion level of the HI in the diet 7.3%); in the HI50 group, the 50% of the dietary protein was replaced by the protein of the HI (inclusion level 14.6%). The hens were housed in the same building in modified cages (800 cm^2^/hen), under controlled temperature (25 °C ± 0.3 °C) and humidity (64% ± 1.2%) conditions. For each group, the hens were distributed into 3 cages (18 hens/cage), and each cage was divided by 2 internal transects into 3 equal areas, to obtain 9 replicates of 6 hens per group. Feed and water were manually distributed, and appropriate separations were placed along the trough and the line of the egg collection to control the feed intake and the egg production per each replicate. The dark:light cycle was 9:15 hours.

### 2.2. Chemical Composition of the Ingredients and Diets

The main protein sources (soybean and insect meal) and the diets were analyzed for the chemical composition according to the AOAC official methods [20]. The metabolizable energy of the diets was calculated according to the NRC estimation procedure [21], while the apparent metabolizable energy of the insect meal used in the present trial was obtained from the studies of De Marco et al. [22]. The data on the amino acids, minerals, and metabolizable energy values of all the ingredients were supplied by the respective producers and used to calculate the correspondent contents in the diets. The amount of protein linked to the acid detergent fiber (ADF) was determined [20]; and only for the insect meal, it was used to estimate the amount of chitin, according to Marono et al. [23] as follows: chitin (%) = ash free ADF (%) − ADF-linked protein (%). The chemical-nutritional characteristics of the two protein sources are reported in Table 1, while the ingredients and chemical-nutritional characteristics of the diets are indicated in Table 2.

### 2.3. Trace/Toxic Elements Determination

The trace elements contained in the protein meals and the toxic elements in the insect meal and in the diets were also determined. The samples were digested in ultrapure 65% HNO_3_ and H_2_O_2_ in a microwave digestion system [24]. Trace elements concentrations were determined by Inductively Coupled Plasma Optical Emission Spectroscopy (ICP-OES) technique using a Perkin Elmer Optima 2100 DV instrument coupled with a CETAC U5000AT. The calibration curve and two blanks were run during each set of analyses, to check the purity of the chemicals. A reference material (CRM DORM-4, National Research Council of Canada (NRC-CNRC), Ottawa (Ontario), Canada) was also included for quality control. All the values of the reference materials were within the certified limits. The instrumental detection limits are expressed as wet weight (w.w.) and were determined following the protocol described by Perkin Elmer ICP application study number 57 [25].

### 2.4. Villus and Crypt Morphometry

At 40 weeks of age, two hens, randomly chosen per replicate (18 per group), were slaughtered, the different digestive tracts were identified, and each of them was excised and weighed.

A tissue sample of the small intestine tracts was rinsed with an iced saline buffer (pH 7) blotted with absorbent paper and divided into three segments, duodenum, jejunum, and ileum.

The intestinal samples (0.5 cm) from duodenum, jejunum, and ileum were fixed by immersion in 4% phosphate-buffered paraformaldehyde for 48 h. The samples were then washed in a phosphate-buffered saline solution, dehydrated in graded ethanol series, and embedded in paraffin according to Vargas et al. [26]. Cross sections (5 µm) at an interval of 200 µm were stained with Mayer’s hematoxylin and eosin and examined under Zeiss Axio Imager.A2 microscope for the histopathological assay. For the morphometric analysis of villus and crypts, ten randomly chosen microscopic fields from each section of the duodenum, jejunum, and ileum were acquired using a microscope combined color digital camera Axiocam 503 (Zeiss, Oberkochen, Germany), and the measurements were performed using the Zen 2.3 lite software.

### 2.5. Brush Border Membrane Enzymes Activity

The Brush Border Membrane (BBM) enzymes were obtained according to Shirazy-Beechey et al. [27] with some modifications as detailed in Messina et al. [28].

The hydrolysis of sucrose and maltose by the mucosal maltase and sucrase-isomaltase (SI) was determined according to Tibaldi et al. [29]. The intestinal alkaline phosphatase (IAP) and γ-glutamyl transpeptidase (γ-GT) activities were determined on the supernatant using two commercial kits (Paramedical, Pontecagnano Faiano, SA, Italy) as indicated by the manufacturer. The total protein concentration was determined according to Bradford [30] (Sigma-Aldrich cat. no. B6916), and bovine serum albumin (Sigma-Aldrich cat. no. 0834) was used as a standard. One unit (U) of enzyme activity corresponded to the amount of enzyme that transforms or hydrolyzes 1 μmole of substrate mL^−1^ min^−1^. The specific enzymatic activity was calculated as U of enzyme activity per mg of protein.

### 2.6. Volatile Fatty Acids (VFAs)

Both the caeca of each slaughtered hen were tied at the ends, separated from the rest of the gastrointestinal tract, put in plastic bags in a pre-warmed thermos (37 °C), and transported in about 1 h to the laboratory. Two aliquots of the caecal content (~5 mL each) were used to measure the VFAs production. Each aliquot was diluted with oxalic acid (1:1, *v*/*v*), and VFAs were determined by a gas chromatography method [31], using an equipment (Thermo-Electron mod. 8000top, FUSED SILICA Gaschromatograph, ThermoElectron Corporation, Rodano, Milan, Italy) with an OMEGAWAX 250 fused silica capillary column (30 m × 0.25 mm × 0.25 mm film thickness), a flame ionization detector (185 °C), the helium as gas carrier (1.7 mL/min), and under isothermal condition (125 °C).

### 2.7. Statistical Analysis

The data were processed by ANOVA using the PROC GLM in SAS [32]. The differences among the groups regarding the VFAs in the caeca, the intestinal morphometry, and enzymatic activity were analyzed by the one-way ANOVA according to the following model: Yij = m + Di + eij, where Y is a single observation, m is the general mean, D is the effect of the diet (i = SBM, HI25 or HI50), and e is the error.

The comparison among the means was performed by the Tukey’s test [32]; in addition, the orthogonal contrast analysis was performed to test the linear and quadratic effect among the means [32].

## 3. Results

### 3.1. Trace/Toxic Elements

The data on the concentration of trace elements of the protein sources are reported in Table 2, and the toxic elements’ concentrations in the HI meal and in the experimental diets are reported in Table 3.

### 3.2. Villus and Crypt Morphometry

Table 4 shows the morphometry traits (villi height, crypt depth, and villi/crypt ratio) for each of the three tracts of the small intestine of the hens according to dietary treatments. In the duodenum, villi height and villi/crypt ratio were higher (*p* < 0.01) in the SBM than in both HI groups. The contrast analysis showed a significant (*p* < 0.01) linear effect for villi height, while the quadratic effect was significant (*p* < 0.01) both for the villi height and villi/crypt. In the jejunum, the villi height and villi/crypt ratio of the hens fed SBM was higher (*p* < 0.01) than that of both HI diets. The contrast analysis showed a significant effect for the linear component for the villi height, villi/crypt (*p* < 0.01), and for the crypt depth (*p* < 0.05). In the ileum, the HI25 group had a higher villi height than the HI50 (*p* < 0.05), and the only significant contrast was a linear effect (*p* < 0.054) for the villi height.

### 3.3. Brush Border Membrane Enzymes Activity

Table 5 reports the activities of brush border enzymes in each of the three tracts of the hen’s small intestine, according to dietary treatments. In the duodenum, the maltase was higher (*p* < 0.05) in the SBM and HI25 than the HI50 group and the contrast analysis indicated a significant linear effect for the maltase and IAP (*p* < 0.05). In the jejunum, the IAP of the SBM was higher than that of both the HI groups and the contrast analysis showed a significant linear effect (*p* < 0.05) for the IAP and γ-GT. In the ileum, the γ-GT showed higher values in the SBM than both the insect meal groups (*p* < 0.05), with a quadratic significant effect (*p* < 0.05).

### 3.4. Volatile Fatty Acids

Table 6 reports the effect of the dietary treatments on the VFAs production in the hen’s caeca. The production of acetate and total VFAs (mmol/L) was higher in the caecal content of the hens fed the HI50 diet than in that of the other groups (*p* < 0.05). The butyrate content was higher (*p* < 0.05) in the HI50 than in the SBM group, while the valerianic acid of the hens fed the SBM diet was higher (*p* < 0.01) than those fed both the HI diets. The contrast analysis showed a significant linear effect (*p* < 0.05) for the isobutyrate, butyrate, isovalerianic, and valerianic acids. 

In Table 6, the VFAs content is also expressed as a percentage of the total VFAs. In this case, the isobutyrate had a higher production in the caeca of the hens fed the HI diets compared to the control (*p* < 0.05), the butyrate in the HI50 group was higher (*p* < 0.05) than the control, while the valerianic acid in control was higher (*p* < 0.01) than both the HI groups. The contrast analysis showed a significant linear effect for butyrate and isovalerianic acids (*p* < 0.05) and for the valerianic acid (*p* < 0.01). For valerianic acid, the quadratic effect was also significant (*p* < 0.01).

## 4. Discussion

The trace minerals content of the insect meals is still poorly investigated, but it is an important goal to formulate appropriate diets for poultry and avoid a possible excessive excretion into the environment. In addition, the knowledge of the potential accumulation of toxic elements is mandatory to guarantee humans and animals safety. A recent study showed that the concentration of many minerals (magnesium, calcium, iodine, iron, sodium, and potassium) in black soldier fly larvae increased linearly as the level of the correspondent mineral in the growing substrate increased, while manganese remained stable in the larvae, despite varying concentrations in the media [17]. The data available in the literature on the mineral concentration of the black soldier fly larvae showed a high variability [7,17,18], and thus it is difficult to compare our results to the available data. The discrepancy of the results is tied to the different kind of substrates used for the insects’ growth but also, for some other elements as Zn, to the technological process following the larvae harvesting. However, the concentrations of Co, Ni, and Se found in the edible insect of our trial were lower than those of the conventional food. The insect meal had a higher amount of Zn, considering the inclusion level, so the only insect meal supplied a daily amount of Zn (331.1 and 662.3 mg/kg, respectively, for HI25 and HI diets) exceeding the correspondent requirements indicated by Hy-Line Brown commercial layers management guide [33] (minimum required 80 mg/kg in a complete diet during the laying period).

Concerning the toxic elements, the concentration of Cd, Pb, and Hg was negligible in all the analyzed samples. The mean value of As was 0.23 mg/kg in the HI meal, approximately comparable to the data reported in the literature and lower than in the complete diet samples.

Compared to the MLs of heavy metals set by the EU Commission [15], Cd, Pb, Hg, and As levels in the diets and insect meal were always lower than the maximum values established for the feeding stuff and feed materials. In fact, the EU regulation establishes the following MLs of heavy metals content in mg/kg (ppm) relative to a feed with a moisture content of 12%: the Cd MLs in the feed materials and complete feed are 2.0 and 0.5 mg/kg, respectively; the Pb MLs in the feed materials and complete feed are 10.0 and 5.0 mg/kg, respectively; the Hg MLs in the feed materials and complete feed are both 0.1 mg/kg; the As MLs in the feed materials and complete feed are both 2 mg/kg.

The inclusion of an insect meal from HI larvae as 25 or 50% substitution of the soybean meal proteins had several effects on the small intestine morphometry and enzymatic activity, as well as on the caecal microbial activity. The morphometry changes mainly occurred in the duodenum and jejunum, but there were also some interesting modifications in the ileum. In general, the villi height decreased as the inclusion level of the insect meal increased in the diet, this is in accordance with the decreased nutrient ileal digestibility recorded in the first part of this trial [12]. The crypt depth was unchanged among the groups, or it tended to decrease (in the jejunum) as the level of the HI increased in the diet. The small intestine is involved in the digestion and absorption of almost all the dietary nutrients [34]: the duodenum digests around 95% of the fats [35]; jejunum digests and absorbs fats, starch, and protein [36,37]; the ileum is mainly involved in water and mineral absorption, but it also digests and absorbs fats, proteins, and starch [34]. The morphological studies of the small intestine are often used to assess its functionality and, in general, an increased villi height is indicative of an improved intestinal function [38]. Another important consideration is that the ileal villi in chickens are smaller and lower than those of the previous tracts of the small intestine, as in hens fed corn-soybean based diets, very little amount of nutrients are available beyond the jejunum [39,40,41]. In the present trial, the height of the ileal villi was lower than that of the duodenum, but higher compared to the jejunum for hens fed both the insect diets. The effect of the diets on the intestinal villi height can be affected by the nutrient digestibility of the diets. As recorded in the first part of this trial [12], the dry matter digestibility of the SBM and the HI25 diets (75.0% and 70.3%, respectively) in hens was higher (*p* < 0.01) than that of the HI50 group (64.3) and the result was mainly attributable to the crude protein digestibility (86.2 vs. 81.1 vs. 76.1%, respectively, for SBM, HI25, and HI50, *p* < 0.01). The low nutrient digestibility in the hens fed the insect meal is tied to the chitin, present in the insect exoskeleton [3]. Thus, our hypothesis is that higher availability of nutrients in the duodenum and jejunum of the hens fed the SBM, increased the intestine functionality, improving the villi height. In insect fed hens, the lower nutrient digestibility induced an increased amount of the potential digestible nutrients in the ileum. Yamauchi et al. [42] stated that an increased load of nutrients deriving directly from the duodenum to the ileum (both for jejunum dissection or different diets) might stimulate the ileal absorptive function, resulting in a compensatory development of the villi. In general, longer villi are the result of activated cell mitosis in the crypts [43]; thus, a larger crypt area indicates a more intensive cell production. In the present trial, the only crypt depth was recorded, and this was unchanged among the groups in the duodenum and ileum tracts. However, the villi height to crypt depth ratio is strongly related to the epithelial cell turnover [44]. In our trial, the cell turnover was higher in the SBM than in the insect meal groups for the duodenum and jejunum, while no differences were observed among the groups in the ileum.

The presence of HI meals in the diet did not affect the activity of both the disaccharases except for the maltase in the duodenum of the hens fed the highest level of HI meal. Recently, Khol et al. [45] showed that the activity of maltase in the small intestine of mallard, chicken, and quail varied depending on the species and, in the mallard, on the intestinal tract. Working with geese, they also demonstrated an effect of the interaction with the protein and the fiber content of the diet, with the highest activity of maltase registered in the Low Protein-Low Fiber group. Likewise, in the present study, the limiting action of the chitin inside the HI meal on the availability of starch during the digestion process led to lower availability of substrate for the maltase activity.

The linear decrease of IAP in the duodenum and jejunum of the hens as the dietary insect meal inclusion increased, shows that the SBM group presents the highest intestinal functionality. Similar results have also been observed by Cutrignelli et al. [46], when the inclusion of HI larvae meal as 50% substitution of soybean meal protein in laying hens decreased the IAP levels in the jejunum and ileum. This enzyme is considered an excellent marker for the crypt-villus differentiation in chicken [47], and, in the present study, the inclusion of HI meal resulted in a negative effect in the jejunum on both the villi/crypt ratio and the IAP specific activity.

The γ-glutamyl transpeptidase plays an essential role in the final digestion and absorption of the dietary proteins being involved in the amino acid transport in the intestine [48,49]. Overall, the effect of the inclusion of HI meal on the activity of the γ-GT in the ileum seems to be in contrast with the increased ileal villi height, while it is in agreement with the weight gain results reported by Bovera et al. [12].

The inclusion of the insect meal in the hens’ diet induced several modifications in the microbial activity in the caeca, as showed by the VFAs production, but the effects were particularly evident with the highest inclusion level (HI50 group). The increased total VFAs production in the latter was mainly due to a higher production of butyrate (+21.5%) and acetate (+13.3%) than in the SBM group, while the valerianic acid decreased in the two groups fed the insect meal. These results completely agree with the finding of Cutrignelli et al. [46], in which the hens fed an HI meal in total replacement of the soybean proteins showed an increased production of butyrate (+62.6%) and acetate (+36.1%) than the control. Similar to our results, Loponte et al. [6] found an increased amount of the total VFAs (+45.6%), acetate (+40.3%), and butyrate (+64.6%) in broilers fed a *Tenebrio molitor* larvae meal as a complete replacement of the soybean meal. The increased activity of the microbial population in the caeca can be related to the chitin level of the HI diet, confirming the hypothesis of Loponte et al. [6] and Cutrignelli et al. [46]. However, another important point emerging from our research is that the chitin needs to be at a sufficient level to act as “prebiotic”, stimulating the intestinal microbial activity. Based on our analysis and taking into account the formula proposed by Marono et al. [23] for the estimation of the insect chitin from the chemical composition, the amount of the chitin in the *H. illucens* larvae meal used in this trial was 6.64% as feed. The feed intake of the hens involved in this trial, and reported by Bovera et al. [12], was 99.97, 97.69, and 101.9 g/day, respectively, for the SBM, HI25, and HI50 groups. Thus, considering the inclusion level in the diets, the HI25 group ingested around 0.47 g/d of chitin, while the HI50 group ingested around 0.99 g/d. Our hypothesis is that the lowest level of chitin is not sufficient to modulate the microbial population activity in the hens. The butyric acid is considered to be the main enterocytes energy source [50], and it is also necessary for the proper development of the Gut-Associated Lymphoid Tissue [51]. It is reported that the VFAs, in general, have a bacteriostatic effect against some enteric bacteria, including *Salmonella typhimurium*, and, in particular, the butyrate is related to the decreased amounts of Enterobacteriaceae in chickens [52]. Thus, both the increases in the total VFAs and butyric acid can improve, through different mechanisms, the health of the hens’ intestine.

Very interesting are the significant changes in the mutual proportions of the butyric, isobutyric, and valerianic acid observed, which might indicate that not only the activity but also the interactions among the different microbial species have been modified. This is in accordance with the findings by Borrelli et al. [53], who observed changes in the gut microbiota of hens fed HI larvae meal. In particular, these authors found a strong correlation between levels of the bacteria strains *Flavonifractor plautii*, *Christensenella minuta*, and *Alkaliphilus transvaalensis* and high production of propionate, butyrate, and total VFAs; these bacteria are the principal contributors to β-N-acetylhexosaminidases and N-acetylglucosamine 6-phosphate deacetylase production, and these enzymes represent the key enzymes responsible for a higher VFAs production.

## 5. Conclusions

The inclusion of an insect meal from HI larvae as 25 or 50% substitution of the proteins from the soybean meal influenced the small intestine morphometry and enzymatic activity, as well as the caecal microbial activity. The SBM group had the highest intestinal functionality, while some compensatory mechanism, probably mediated by the chitin, led to a positive increase of the VFAs and butyrate in the HM50 diet with potential positive effects on the gut healthiness. However, considering the results of Bovera et al. [12], to which these results are strongly linked, it is possible to conclude that the 25% soybean protein replacement with the *Hermetia illucens* larvae meal in the diet of laying hens is more suitable and closer to the optimal level than 50% replacement. Finally, the levels of Cd, Pb, Hg, and As in the diets and insect meal were always lower compared to MLs of heavy metals set by the EU Commission for the feeding stuff and feed materials. This latter aspect provides important information on the safe use of these alternative ingredients in animal feeding.

## Figures and Tables

**Table 1:** Proximate composition, mineral, and essential amino acid composition (% as fed) of the *Hermetia illucens* larvae defatted meal and soybean meal.

	*Hermetia illucens* Larvae Meal	Soybean Meal
Proximate composition		
Dry matter	92.7	90.0
Crude protein	55.6	43.4
Ether extract	8.34	1.1
ADF	11.5	5.9
ADF-linked protein	4.86	1.78
Ash	7.8	6.0
Mineral composition		
Ca ^1^	6.47	2.83
Total P ^1^	0.90	0.57
Na ^1^	0.12	0.16
Trace element composition, mg/kg		
Cr	0.21	0.24
Co	0.01	0.11
Cu	3.20	16.3
Mn	45.1	32.5
Fe	10.7	11.8
Ni	0.10	2.50
Se	0.05	0.005
Zn	453.6	54.23
Essential Amino Acid composition		
Lysine ^1^	4.12	2.92
Methionine ^1^	1.09	0.61
Methionine + Cystine ^1^	1.32	1.33
Isoleucine ^1^	2.97	2.30
Tryptophan ^1^	0.30	0.73
Valine ^1^	5.02	2.11
Threonine ^1^	2.32	1.74

ADF: Acid Detergent Fiber; ^1^ obtained by the producers.

**Table 2:** Ingredients and chemical characteristics of the three diets: control (SBM), HI25, and HI50.

	SBM	HI25	HI50
**Ingredients, g/kg**			
Maize grain	605.5	597.5	630.5
Soybean meal	265	200	95
Insect meal	-	73	146
CaCO_3_ grains	80	80	80
Vegetable oil	10	10	-
MinVit *	10	10	10
Methionine	2.5	2.5	2.5
Monocalcium phosphate	5	5	5
Celite	20	20	20
Salt	2	2	2
**Chemical-nutritional characteristics**			
Dry matter ^1^, %	91.53	91.39	91.62
Crude protein ^1^, %	16.45	16.32	17.03
Ether extract ^1^, %	3.17	3.61	4.06
NDF ^1^, %	10.38	11.29	12.49
ADF ^1^, %	5.85	5.90	5.67
ADL ^1^, %	2.67	2.94	2.29
Lysine ^2^, %	0.86	0.97	1.00
Methionine ^2^, %	0.53	0.58	0.61
Metabolizable Energy ^2^, kcal/kg	2832.3	2845.2	2842.2

SBM: soybean meal-based diet; HI25: diet including HI as 25% of replacement of the soybean meal protein; HI50: diet including HI as 50% of replacement of the soybean meal protein; NDF: Neutral Detergent Fiber; ADF: Acid Detergent Fiber; ADL: Acid Detergent Lignin; ^1^: determined according to AOAC (2004); ^2^: calculated according to NRC (1994); * Provided per kilogram: vitamin A (retinyl acetate) 20,000 IU, vitamin D3 (cholecalciferol) 6000 IU, vitamin E (dl-α-tocopheryl acetate) 80 IU, vitamin B1 (thiamine monophosphate) 3 mg, vitamin B2 (riboflavin) 12 mg, vitamin B6 (pyridoxine hydrochloride) 8 mg, vitamin B12 (cyanocobalamin) 0.04 mg, vitamin K3 (menadione) 4.8 mg; vitamin H (d biotin) 0.2 mg, vitamin PP (nicotinic acid) 48 mg, folic acid 2 mg, calcium pantothenate 20 mg, manganous oxide 200 mg, ferrous carbonate 80 mg, cupric sulphate pentahydrate 20 mg, zinc oxide 120 mg, basic carbonate monohydrate 0.4 mg, anhydrous calcium iodate 2 mg, sodium selenite 0.4 mg, choline chloride 800 mg, 4-6-phytase 1800 FYT, D.L. methionine 2600 mg, canthaxanthin 8 mg.

**Table 3:** Toxic elements’ content (mg/kg) in the insect meal (HI meal) and in the three diets (SBM, HI25, and HI50).

	HI Meal	SBM	HI25	HI50
As	0.23	0.95	0.86	0.81
Cd	0.06	0.001	-	0.007
Pb	0.03	-	-	-
Hg	0.01	-	-	-

**Table 4:** Effect of the dietary treatments on the villi height and crypt depth of the different small-intestine tracts.

	SBM	HI25	HI50	RMSE	*p*-Values		
					ANOVA	Contrast Analysis	
						Linear	Quadratic
	Duodenum						
Villi height	1394 ^A^	1031 ^B^	1006 ^B^	117.5	<0.0001	<0.0001	<0.0001
Crypt depth	350.8	381.3	395.8	90.56	0.4739	0.2345	0.8059
Villi/crypt	3.99 ^A^	2.69 ^B^	2.90 ^B^	0.79	0.0006	0.0018	0.1023
	Jejunum						
Villi height	1149 ^A^	825 ^B^	790 ^B^	114.2	<0.0001	<0.0001	<0.0001
Crypt depth	273.3 ^b^	275.5 ^b^	315.9 ^a^	26.29	0.0461	0.0329	0.2684
Villi/crypt	4.27 ^A^	3.09 ^B^	2.68 ^B^	0.84	<0.0001	<0.0001	0.1285
	Ileum						
Villi height	1006 ^ab^	1013 ^a^	843 ^b^	161.2	0.0317	0.0162	0.1541
Crypt depth	321.3	335.9	328.7	32.11	0.8308	0.7601	0.6151
Villi/crypt	3.31	3.12	2.77	0.36	0.2351	0.0918	0.7821

SBM: soybean meal group; HI25 and HI50: HI groups in which the soybean protein was replaced by 25% and 50% of the HI larvae meal protein, respectively. ^A,B^: *p* < 0.01; ^a,b^: *p* < 0.05; RMSE: root mean square error.

**Table 5:** The specific activity of the maltase, sucrose-isomaltase (SI), intestinal alkaline phosphatase (IAP), γ-glutamyltransferase (γGT) measured in the different digestive tracts of the hens fed the test diets over 21 weeks.

	SBM	HI25	HI50	RMSE	*p*-Values		
					ANOVA	Contrast Analysis	
						Linear	Quadratic
	Duodenum						
Maltase (U)	70.46 ^a^	74.96 ^a^	50.82 ^b^	19.47	0.0498	0.0465	0.1044
SI (U)	12.12	13.02	11.54	3.49	0.7001	0.7422	0.4419
IAP (U)	4.39	3.87	2.75	1.15	0.3165	0.0422	0.7479
γGT (mU)	129.4	159.2	119.9	59.9	0.2888	0.5582	0.1457
	Jejunum						
Maltase (U)	71.39	69.94	76.77	16.97	0.7025	0.5325	0.5799
SI (U)	17.49	14.65	16.21	4.97	0.5308	0.6118	0.3194
IAP (U)	5.48 ^a^	3.07 ^b^	2.87 ^b^	2.23	0.0436	0.0295	0.2680
γGT (mU)	206.4	199.1	154.9	70.42	0.3064	0.0435	0.5515
	Ileum						
Maltase (U)	77.33	55.92	64.19	22.25	0.1780	0.2505	0.1385
SI (U)	17.34	14.51	17.20	3.90	0.2840	0.9445	0.1172
IAP (U)	3.29	3.09	3.06	1.35	0.9351	0.7379	0.8899
γGT (mU)	145.0 ^a^	94.09 ^b^	132.73 ^ab^	37.0	0.0307	0.513	0.0108

SBM: soybean meal group; HI25 and HI50: HI groups in which the soybean protein was replaced by 25% and 50% of the HI larvae meal protein, respectively. ^a,b^: *p* < 0.05; RMSE: root mean square error.

**Table 6:** Volatile fatty acids in the caecal content of the hens fed the test diets over 21 weeks.

	SBM	HI25	HI50	RMSE	*p*-Values		
					ANOVA	Contrast Analysis	
						Linear	Quadratic
	mmol/L						
Acetate	58.24 ^b^	57.54 ^b^	65.99 ^a^	6.59	0.0242	0.1565	0.3406
Propionate	20.62	20.31	21.42	2.50	0.9169	0.7718	0.7735
Isobutyrate	1.78	2.11	2.34	0.17	0.1442	0.0107	0.8381
Butyrate	7.25 ^b^	7.72 ^ab^	8.81 ^a^	0.59	0.0125	0.0326	0.6576
Isovalerianic	2.78	3.00	3.42	0.25	0.1274	0.0453	0.7919
Valerianic	4.38 ^A^	2.99 ^B^	3.28 ^B^	0.31	0.0021	0.0176	0.3777
Total VFA	95.06 ^b^	93.67 ^b^	105.0 ^a^	6.69	0.0392	0.5826	0.4620
	% total VFAs						
Acetate	61.27	61.43	62.85	5.38	0.8571	0.5743	0.9914
Propionate	21.69	21.68	20.40	2.54	0.8092	0.6036	0.7088
Isobutyrate	1.87 ^b^	2.25 ^a^	2.22 ^a^	0.15	0.0212	0.1375	0.3203
Butyrate	7.62 ^b^	8.24 ^ab^	8.39 ^a^	0.53	0.0456	0.0289	0.8072
Isovalerianic	2.92	3.20	3.26	0.12	0.0953	0.0423	0.8112
Valerianic	4.61 ^A^	3.19 ^B^	3.12 ^B^	0.31	<0.0001	<0.0001	0.0014

SBM: soybean meal group; HI25 and HI50: HI groups in which the soybean protein was replaced by 25% and 50% of the HI larvae meal protein, respectively. ^A,B^: *p* < 0.01; ^a,b^: *p* < 0.05; RMSE: root mean square error.
